# Supplementary material for: Proteomic, Biochemical, and Morphological Analyses of the Effect of Silver Nanoparticles Mixed with Organic and Inorganic Chemicals on Wheat Growth
Source: Cells. 2022 May 7;11(9):1579. doi: 10.3390/cells11091579 (PMC9104970; doi:10.3390/cells11091579)
Supplement: Supplementary file 1 [file cells-11-01579-s001.zip › rev Suplemental Table S1.pdf]

Table S1. Experimental procedure of gel-free/label-free proteomics used in this research

Title	Methods
Protein Enrichment, Reduction, Alkylation, and Digestion	Protein extracts (100 µg) were adjusted to a final volume of 100 µL. Methanol (400 µL) was added to each sample and mixed before addition of 100 µL of chloroform and 300 µL of water. After mixing and centrifugation at 20,000 x g for 10 min to achieve phase separation, the upper phase was discarded and 300 µL of methanol was added to the lower phase, and then centrifuged at 20,000 x g for 10 min. The pellet was collected as the soluble fraction (Komatsu et al., 2013). Proteins were resuspended in 50 mM NH_4HCO_3, reduced with 50 mM dithiothreitol for 30 min at 56°C, and alkylated with 50 mM iodoacetamide for 30 min at 37°C in the dark. Alkylated proteins were digested with trypsin and lysyl endopeptidase (Wako, Osaka, Japan) at a 1:100 enzyme/protein ratio for 16 h at 37°C. Peptides were desalted with MonoSpin C18 Column (GL Sciences, Tokyo, Japan). Peptides were acidified with 1% trifluoroacetic acid and analyzed by nano-liquid chromatography (LC) and mass spectrometry (MS)/MS.
Protein Identification using Nano LC-MS/MS	The peptides were loaded onto the LC system (EASY-nLC 1000; Thermo Fisher Scientific, San Jose, CA, USA) equipped with a trap column (Acclaim PepMap 100 C18 LC column, 3 µm, 75 µm ID x 20 mm; Thermo Fisher Scientific) equilibrated with 0.1% formic acid and eluted with a linear acetonitrile gradient (0-35%) in 0.1% formic acid for 120 min at a flow rate of 300 nL/min. The eluted peptides were loaded and separated on the column (EASY-Spray C18 LC column, 3 µm, 75 µm ID x 150 mm; Thermo Fisher Scientific) with a spray voltage of 2 kV (Ion Transfer Tube temperature: 275°C). The peptide ions were detected using MS (Orbitrap Fusion ETD MS; Thermo Fisher Scientific) in the data-dependent acquisition mode with the installed Xcalibur software (version 4.0; Thermo Fisher Scientific). Full-scan mass spectra were acquired in the MS over 375 - 1,500 m/z with a resolution of 120,000. The most intense precursor ions were selected for collision-induced fragmentation in the linear ion trap at normalized collision energy of 35%. Dynamic exclusion was employed within 60 sec to prevent repetitive selection of peptides (Zhang et al., 2009).
MS Data Analysis	The MS/MS searches were carried out using MASCOT (version 2.6.1, Matrix Science, London, U.K.) and SEQUEST HT search algorithms against Triticum aestivum (SwissProt TaxID=4565_and_subtaxonomies) (version 2017-07-05) protein database that the size of the protein database is SwissProt=370, TrEBML=145,221, and total=145,591, using Proteome Discoverer (PD) 2.2 (version 2.2.0.388; Thermo Scientific). The workflow for both algorithms included spectrum files RC, spectrum selector, MASCOT, SEQUEST HT search nodes, percolator, ptmRS, and minor feature detector nodes. Oxidation of methionine was set as a variable modification and carbamido methylation of cysteine was set as a fixed modification. MS and MS/MS mass tolerances were set to 10 ppm and 0.6 Da, respectively. Trypsin was specified as protease and a maximum of one missed cleavage was allowed. Target-decoy database searches were used for the calculation of false discovery rate (FDR) and FDR for peptide identification was set at 1%.

Differential Analysis of Proteins using MS Data	Label-free quantification was performed with PD 2.2 using precursor ions quantifiler nodes. For differential analysis of the relative abundance of peptides and proteins between samples, the free available software Perseus (version 1.6.0.7; Max Planck Institute of Biochemistry, Martinsried, Germany) (Tyanova et al., 2016) was used. Proteins and peptides abundances were transferred into log2 scale. Three biological replicates of each sample were grouped and a minimum of 3 valid values were required in at least one group. Normalization of the abundances was performed to subtract the median of each sample. Missing values were inputted based on a normal distribution (width = 0.3, down-shift = 1.8). Significance was assessed using t-test analysis. Principal component analysis (PCA) was performed with Perseus. The gene functional annotations and protein categorization was analyzed using MapMan bin codes (Usadel et al., 2005).

Komatsu, S.; Han, C.; Nanjo, Y.; Altaf-Un-Nahar, M.; Wang, K.; He, D.; Yang, P. Label-free quantitative proteomic analysis of abscisic acid effect in early-stage soybean under flooding. *J Proteome Res* **2013,** *12*, 4769-4784.

Zhang, Y.; Wen, Z.; Washburn, M.P.; Florens, L. Effect of dynamic exclusion duration on spectral count based quantitative proteomics. *Anal Chem* **2009,** *81,* 6317-6326.

Tyanova, S.; Temu, T.; Siniteyn, P.; Carlson, A.; Hein, Y.; Gieger, T.; Mann, M.; Cox, J. The Perseus computational platform for comprehensive analysis of proteomics data. *Nat Methods* **2016,** 13, 731-740.

Usadel, B.; Nagel, A.; Thimm, O.; Redestig, H.; Blaesing, O.E.; Rofas, N. P.; Selbig, J.; Hannemann, J.; Piques, M. C.; Steinhauser, D.; Scheible, W. R.; Gibon, Y.; Morcuende, R.; Weicht, D.; Meyer, S.; Stitt, M. Extension of the visualization tool MapMan to allow statistical analysis of arrays, display of corresponding genes and comparison with known responses. *Plant Physiol* **2005,** *138,* 1195-1204.
