# Supplementary material for: Proteomic, Biochemical, and Morphological Analyses of the Effect of Silver Nanoparticles Mixed with Organic and Inorganic Chemicals on Wheat Growth
Source: Cells. 2022 May 7;11(9):1579. doi: 10.3390/cells11091579 (PMC9104970; doi:10.3390/cells11091579)
Supplement: Supplementary file 1 [file cells-11-01579-s001.zip › rev2 Suplemental Table S3.pdf]

Table S3. List of significantly changed proteins in wheat roots treated with AgNPs, nicotinate, and KNO_3 compared to untreated plant.

Log2 (Fold Change)	Accession	Description	MW (kDa)	pI	LOG *P*Value	MapMan function
4.1531	A0A1D6S6K6	RNA-binding domain-containing protein	21.6	9.33	1.3046	not assigned
2.5429	Q9SQ58	Starch synthase	66	6.83	1.6040	major CHO metabolism
2.4902	W5ANZ2	Nucleosome/chromatin assembly factor	13	8.25	1.5989	RNA
1.9766	W5FKR1	MOSC domain-containing protein 2	35.6	5.47	1.3216	not assigned
1.9709	A0A077RYT2	G-proteins	23.2	5.31	2.0418	signalling.
1.8830	W5GSR7	Glutamyl-tRNA(Gln) amidotransferase	60.6	6.42	3.1489	tetrapyrrole synthesis.
1.5827	A0A1D5ZQH0	UPI0003D5684F	10.8	10.08	2.1853	N/A
1.4154	A0A1D5SDG4	Predicted protein	47.4	6.37	1.3921	protein.targeting.
1.2905	A0A096UW30	Uncharacterized protein C16G5.07c	40.1	7.49	1.4294	not assigned
1.2218	A0A1D6AR09	Predicted protein	27.7	8.6	1.4527	not assigned
1.1719	A0A1D6AQK5	Uncharacterized protein	16.6	7.18	1.7471	not assigned
1.1602	A0A1D6CLF4	Predicted protein	30.5	9.52	2.2144	protein.assembly
1.1576	A0A1D5VED9	Uncharacterized protein	63.1	5.36	1.6057	not assigned.
1.1289	A0A1D5UQ46	Uncharacterized protein	43.6	7.78	1.7705	not assigned.
1.1013	A0A1D5U2X7	Glutathione peroxidase	19.3	7.84	1.9192	redox.
1.0624	A0A1D5RWJ9	Predicted protein	47.3	6.58	1.7070	protein.targeting
1.0446	A0A1D5T3J1	pyruvate dehydrogenase E3	52.9	7.96	1.4967	TCA
1.0112	W5A446	proline rich family	23.3	4.55	3.4421	not assigned.
0.9045	A0A1D5SPQ1	proline rich family	23.2	4.49	1.7132	not assigned.
0.8908	A0A1D6B3R4	homoserine kinase	37.9	8.57	1.8444	amino acid metabolism
0.8560	A0A1D5X2D4	Ferredoxins and Rieske domain	29.9	5.67	2.5173	misc.
0.8507	A0A1D5YL67	glyceraldehyde-3-phosphate dehydrogenase	46.9	6.47	2.1841	glycolysis
0.8351	A0A1D5XGM1	catalase	56.8	7.02	1.5226	redox
0.8231	A0A1D5UZ55	Uncharacterized protein	27.2	8.97	2.9630	not assigned
0.7474	A0A1D5YIN9	polypeptide subunits	35	9.94	2.1478	glycolysis
-0.6253	W5FL28	Lipoxygenase	105.4	6.11	2.3587	hormone metabolism
-0.6621	C6K7G3	Lipoxygenase	96.5	6.65	3.2198	hormone metabolism
-0.6982	A0A0C4BJG7	Glutamine synthetase	38.7	5.72	1.7444	N-metabolism
-0.7649	A0A1D5WPA9	aspartate protease	44.5	8.1	1.5657	protein.degradation.
-0.7843	A0A1D5THX4	glycosyl hydrolase family 5	64.7	6.32	2.2351	misc.

-0.8113	A0A1D6RGV7	glutathione S transferases	24.9	5.81	1.8697	redox
-0.8148	A0A1D5WAN2	Orphan family	92.1	5.47	1.6018	RNA
-0.8399	Q6RUJ2	Glutamine synthetase	38.7	5.58	4.1135	N-metabolism
-0.9085	A0A1D5VLU6	Uncharacterized protein	41.3	5.38	2.4205	not assigned
-0.9164	B2ZGL3	Plastid acetyl-CoA carboxylase	254.9	6.13	2.7962	lipid metabolism
-0.9202	A0A1D6DH60	glycosyltransferase	53.2	6.21	2.3466	misc.
-0.9207	A0A1D5U520	glycolate oxydase	47.8	9.04	2.1154	glycolysis
-0.9619	D5MTE2	glycosyltransferase	49.7	5.8	1.6936	misc.
-1.0086	A0A077RS68	late embryogenesis abundant	42.5	5.22	1.7809	development.
-1.0156	A0A1D6DIS2	late embryogenesis abundant	45.6	8.66	2.7720	not assigned
-1.0174	W5FUL1	asparagine synthetase	71.9	7.12	3.3894	amino acid metabolism
-1.0220	A0A1D6CAY4	Uncharacterized protein	39.5	8.1	2.2531	not assigned
-1.0587	A0A1D5SSN1	Uncharacterized protein	52.2	7.12	2.8195	secondary metabolism
-1.0680	A0A1D5UE08	PDC	64.9	6.32	1.8328	fermentation.
-1.0998	W5GZ16	aspartate family.lysine	115.5	5.87	3.5646	amino acid metabolism.
-1.1062	A0A1D5XSR2	Xyloglucan endotransglucosylase	33.5	7.01	1.9681	cell wall
-1.1518	A0A1D6CKD4	Uncharacterized protein	24.6	4.79	2.0428	stress.biotic
-1.1590	A0A077RVE4	Uncharacterized protein	28.9	4.84	2.3276	not assigned
-1.2332	A0A1D6BH63	Uncharacterized protein	29.9	5.02	2.8373	stress.abiotic.
-1.2399	W5H7W2	Dihydroflavonol-4-reductase	35.1	6.43	2.9502	secondary metabolism
-1.2553	Q41542	xyloglucan endotransglucosylase	33.4	7.01	1.9645	cell wall.
-1.2968	P30569	EC protein I/II	7.7	7.59	1.9529	metal handling.
-1.3025	A0A1D6BE91	glutathione S transferases	24.8	5.77	2.3181	redox
-1.3693	A0A1D5SZ44	late embryogenesis abundant	22	8.91	2.3281	development.
-1.3708	A0A1D6ACN8	allene oxidase cyclase	25.7	8.62	3.0017	hormone metabolism.
-1.4606	A0A1D5WFR9	peroxidase	38.2	7.08	2.2699	redox
-1.4866	A0A1D6BLA1	Uncharacterized protein	39.6	8.27	2.3366	not assigned
-1.5192	A0A1D5U5D9	amine oxidase	85.7	7.08	3.8945	misc.
-1.5442	A0A1D6ARD7	Uncharacterized protein	14.6	8.72	2.8543	N/A
-1.5471	W5GY26	12-Oxo-PDA-reductase	40.6	6.62	2.7611	hormone metabolism.
-1.5816	A0A1D6BXX6	glutathione S transferases	26.6	5.5	3.0221	redox
-1.6378	A0A1D6CVN2	Polyadenylate-binding protein	70.9	6.95	2.5851	RNA
-1.6883	A0A1D5ZF40	MADS box transcription factor family	70.4	7.01	3.3134	RNA
-1.6909	A0A1D6BB05	Transmembrane 9 superfamily member	72.2	7.42	3.0705	not assigned
-1.6918	A0A1D6C4X1	carbonic anhydrases	32.1	6.25	3.8194	TCA

-1.7481	A0A1D6AEQ8	Predicted protein	47.2	5.94	2.5495	stress.abiotic.
-1.8656	A0A1D5XRM8	Uncharacterized protein	15.7	9.57	3.9836	not assigned.
-1.9147	W5FCA4	Actin depolymerization facto	15.8	4.82	2.3572	cell.organisation
-1.9154	A0A1D6BII6	Uncharacterized protein	81.2	7.43	3.3765	not assigned.
-1.9377	A0A0H4MAT1	Dehydrin	13.9	8.87	2.2928	stress.abiotic.
-1.9690	A0A1D5T629	Predicted protein	47.8	7.02	2.2288	not assigned.unknown
-1.9843	A0A1D5TK55	G-proteins	178.5	5.81	2.5288	signalling.
-2.0122	A0A1D6A238	Uncharacterized protein	65.5	5.26	2.0622	cell.organisation
-2.0275	A0A1D5V489	Casein kinase I-like	52.9	9.74	2.5788	protein.PTM
-2.0403	A0A1D5WKE9	pyrimidine.UMP Synthase	51.1	6.54	1.8803	nucleotide
-2.0784	A0A1D6RTG2	Uncharacterized protein	11.8	8.41	2.5648	N/A
-2.0821	A0A0F7G9F3	WSI18 protein	22.7	7.18	1.8418	development.
-2.4136	A0A1D5X5E7	Thaumatin-like protein TLP8	23.4	7.27	2.4462	stress.biotic
-2.4753	A0A023W4F1	Endochitinase	33.4	7.17	2.4137	stress.biotic
-2.4962	A0A1D5UH99	Uncharacterized protein	29	8.5	1.9768	stress.abiotic
-2.6644	A0A1D5WVH9	late embryogenesis abundant	43.2	5.53	1.7742	development.
-2.7042	A0A077RSI2	Uncharacterized protein	7.5	7.74	1.9215	not assigned
-2.7322	A0A1D5TMQ4	hydroxypyruvate reductase	35.3	6.62	2.0284	glycolysis
-2.9407	A0A023W594	Endochitinase	33.5	7.43	2.0550	stress.biotic
-3.1925	A0A1D6CMV8	Uncharacterized protein	39.6	8.27	4.2445	not assigned
-3.2818	Q4JH28	glucan endo-1,3-beta-glucosidase	35.3	8.48	3.0818	misc.
-3.5183	A0A1D5X1Y2	Thaumatin-like protein	21.7	8.15	1.9979	stress.biotic
-3.8302	A0A1D5UMP3	Chitinase IV	28.6	4.77	1.7457	stress.biotic
-3.9589	O64393	Wheatwin-2	15.9	7.94	2.5250	stress.abiotic
-4.0004	A0A1D5XWK3	asparagine synthetase	66.1	6.49	2.4808	amino acid metabolism

PTM, post translational modification
