# Supplementary material for: Proteomic, Biochemical, and Morphological Analyses of the Effect of Silver Nanoparticles Mixed with Organic and Inorganic Chemicals on Wheat Growth
Source: Cells. 2022 May 7;11(9):1579. doi: 10.3390/cells11091579 (PMC9104970; doi:10.3390/cells11091579)
Supplement: Supplementary file 1 [file cells-11-01579-s001.zip › rev2 Supplemental Figures.pdf]

A

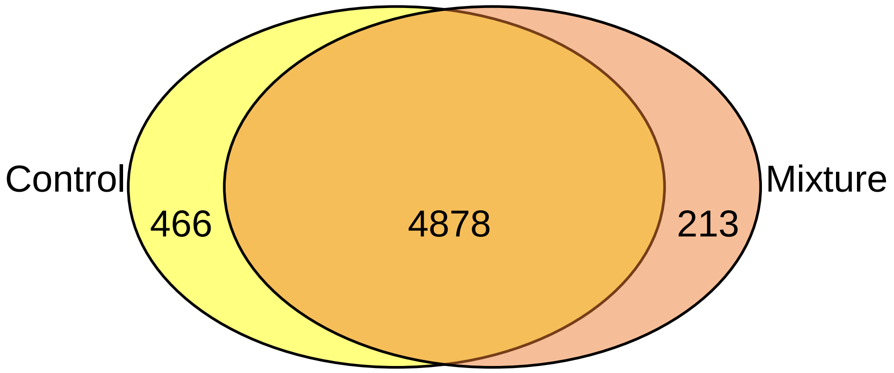

Exclusive		Total	Description
	466	5344	Control
	213	5091	Mixture
	4878	4878	Control/Mixture
Sum	5557		

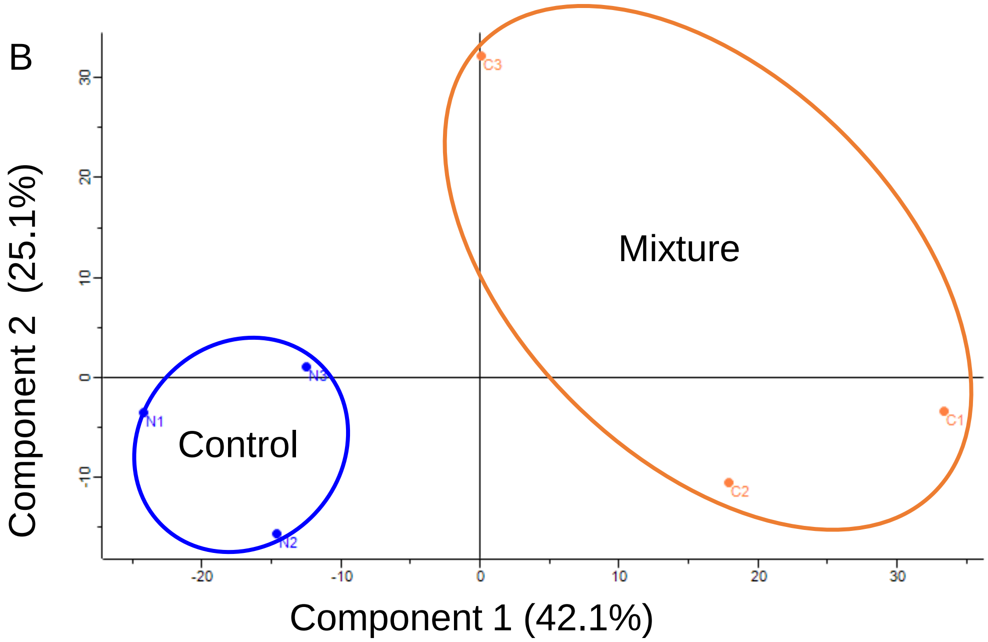

Figure S1. Venn diagram of proteomics results and overview of total proteomic data from 6 samples of wheat root based on PCA. A) Venn diagram of proteomics results. Numbers of figure show protein numbers. B) Overview of total proteomic data from 6 samples of wheat root based on PCA. The blue color and orange color show control group and mixture group, respectively.

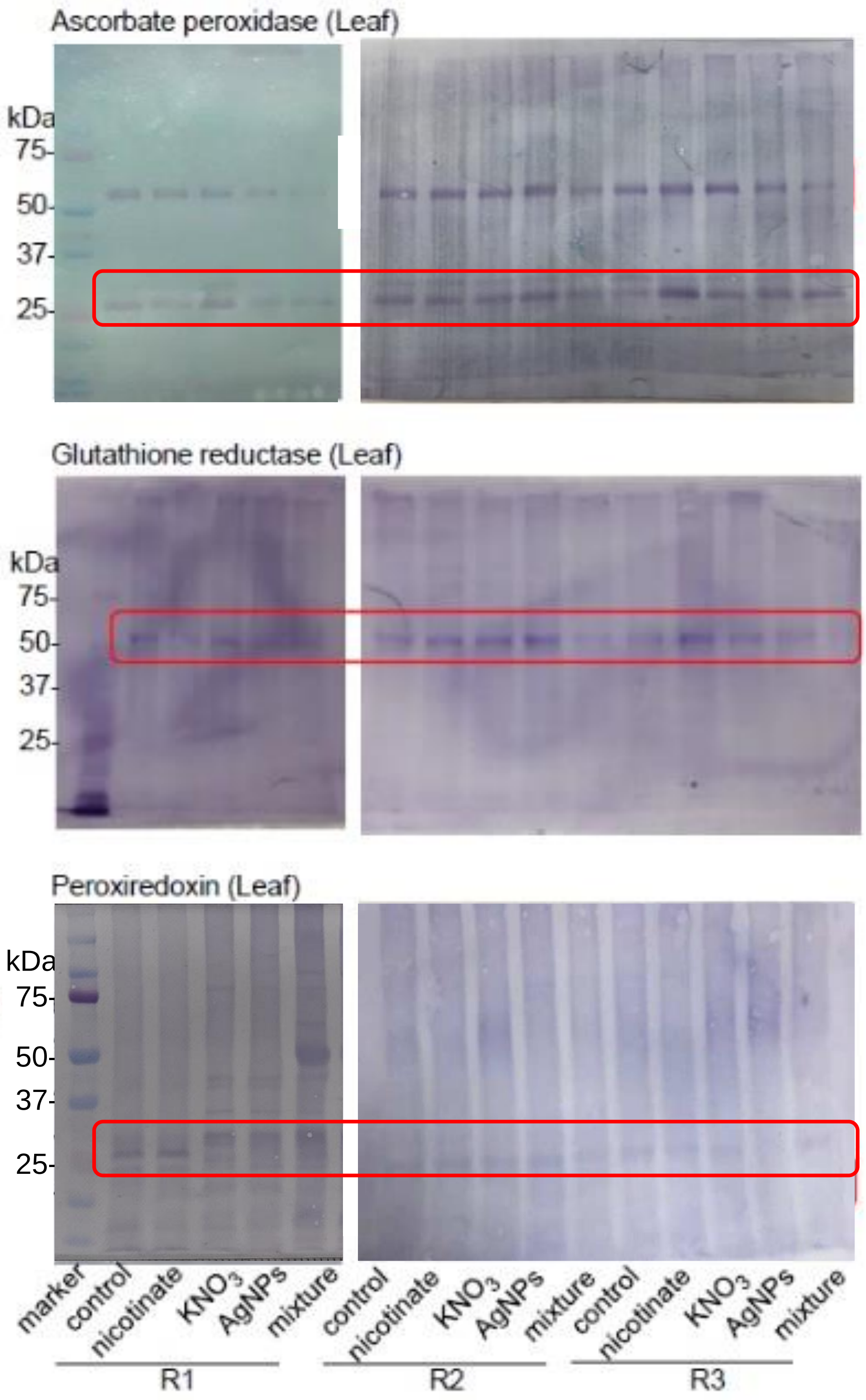

Figure S2. Blots of the entire membrane with anti-ascorbate peroxidase, glutathione reductase, and peroxiredoxin antibodies, which were used in Figure 4 Leaf.

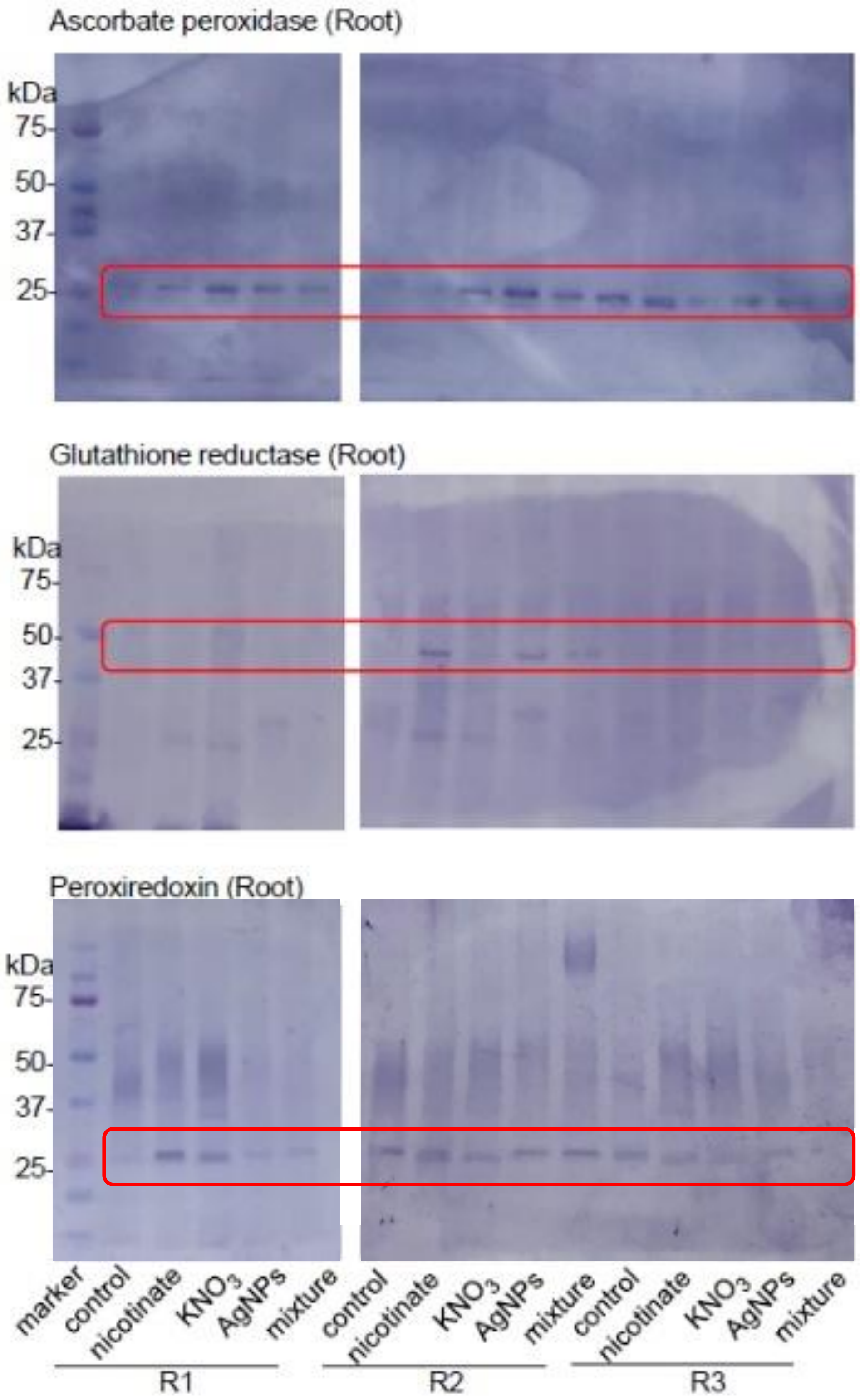

Figure S3. Blots of the entire membrane with anti-ascorbate peroxidase, glutathione reductase, and peroxiredoxin antibodies, which were used in Figure Root.

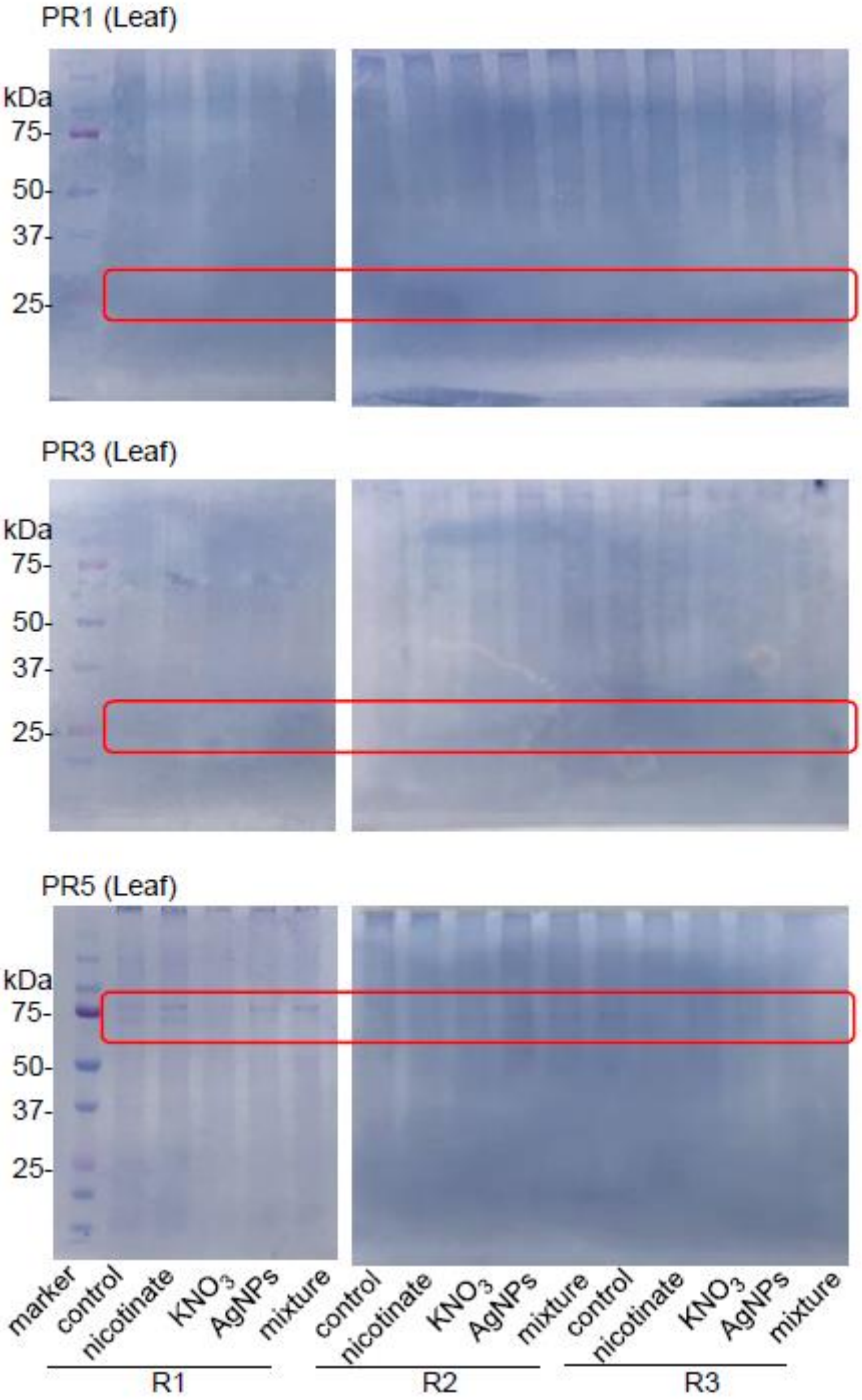

Figure S4. Blots of the entire membrane with anti-PR1, PR3, and PR5, which were used in Figure 5 Leaf.

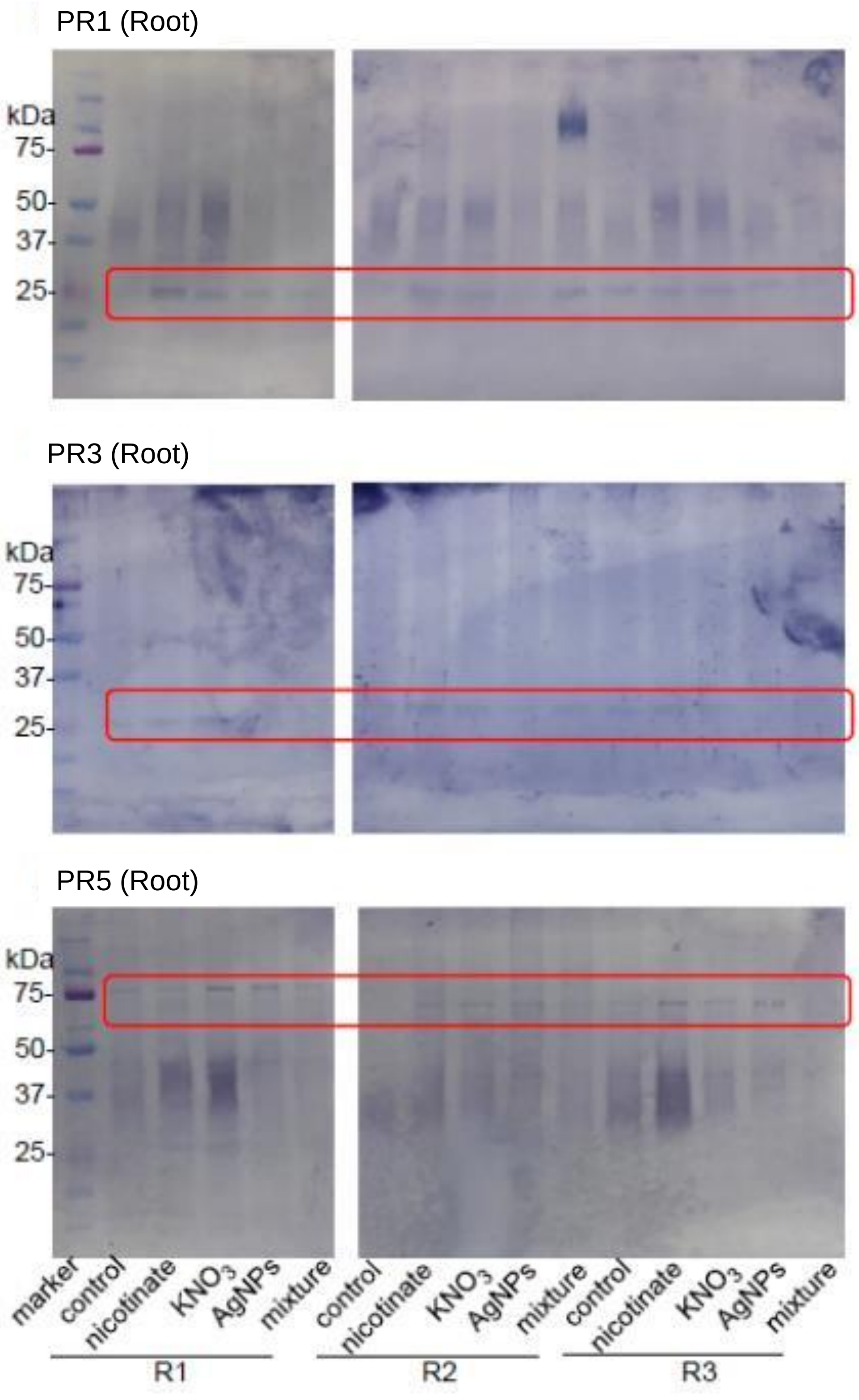

Figure S5. Blots of the entire membrane with anti-PR1, PR3, and PR5, which were used in Figure 5 Root.
